# Frequency and Interrelations of Risk Factors for Chronic Low Back Pain in a Primary Care Setting

**DOI:** 10.1371/journal.pone.0004874

**Published:** 2009-03-16

**Authors:** Marie-Martine Lefevre-Colau, Fouad Fayad, François Rannou, Jacques Fermanian, Fernand Coriat, Yann Mace, Michel Revel, Serge Poiraudeau

**Affiliations:** 1 Department of Physical Medicine and Rehabilitation, Corentin-Celton Hospital (AP-HP), Paris Descartes University, Issy-les-Moulineaux, France; 2 Department of Physical Medicine and Rehabilitation, Cochin Hospital (AP-HP), Paris Descartes University, Paris, France; 3 Department of Biostatistics, Necker Hospital (AP-HP), Paris Descartes University, Paris, France; 4 Laboratoire Sanofi-Aventis, Paris, France; 5 Department of Rheumatology, Hotel Dieu de France, Saint-Joseph University, Beirut, Lebanon; University of Otago, New Zealand

## Abstract

**Introduction:**

Many risk factors have been identified for chronic low back pain (cLBP), but only one study evaluated their interrelations. We aimed to investigate the frequency of cLBP risk factors and their interrelations in patients consulting their general practitioners (GPs) for cLBP.

**Methods:**

A cross-sectional, descriptive, national survey was performed. 3000 GPs randomly selected were asked to include at least one patient consulting for cLBP. Demographic, clinical characteristics and the presence of cLBP risk factors were recorded. The frequency of each cLBP risk factor was calculated and multiple correspondence analysis (MCA) was performed to study their interrelations.

**Results:**

A total of 2068 GPs (68.9%) included at least 1 patient, for 4522 questionnaires analyzed. In the whole sample of patients, the 2 risk factors most commonly observed were history of recurrent LBP (72.1%) and initial limitation of activities of daily living (66.4%). For working patients, common professional risk factors were beliefs, that LBP was due to maintaining a specific posture at work (79.0%) and frequent heavy lifting at work (65.5%). On MCA, we identified 3 risk-factor dimensions (axes) for working and nonworking patients. The main dimension for working patients involved professional risk factors and among these factors, patients' job satisfaction and job recognition largely contribute to this dimension.

**Discussion:**

Our results shed in light for the first time the interrelation and the respective contribution of several previously identified cLBP risk factors. They suggest that risk factors representing a “work-related” dimension are the most important cLBP risk factors in the working population.

## Introduction

General practitioners (GPs) are often consulted for low back pain (LBP). The point prevalence of LBP is reported to be about 15% to 30% in the Western world [1]. For about 6% to 10% of patients, the disease may recur or become chronic and the demand on the health-care system is great and costly [2]–[4]. These patients are also a cause of major disability and absence from work [5], [6]. Fewer than half of individuals disabled for longer than 6 months return to work, and after 2 years of absence from work, the return-to-work rate is close to zero [2], [7]. Moreover, back pain is the most common chronic illness in subjects younger than 65 years [1], [2], [8].

Early identification of risk factors for chronic LBP (cLBP) is important in understanding, and with hope, preventing the progression to chronic disease and disability.

Many studies in Western industrialized countries have attempted to identify risk factors for LBP [2], [9], [10], with a good evidence of relation between cLBP and history of LBP (including pain severity, duration, disability, leg pain, related sick leave and history of spinal surgery), low level of job satisfaction and poor general health [11]–[20]. Only moderate evidence exists for a relation between cLBP and psychosocial factors such as employment status, amount of wages, workers' compensation, and depression [11], [13], [15], [21]–[28] or physical factors such as lifting time per day and work posture [10], [13], [14].

The literature on risk factors for cLBP is abundant with numerous prospective studies done on relatively small samples of patients assessing only a specific category of cLBP risk factors. Moreover, the major drawback in prospective and cross-sectional studies of cLBP risk factors is the use of simplistic methodological approach without considering the interrelations of the known risk factors. These studies do not allow for analyzing the structure of the existing relations between risk factors and discovering the underlying dimensions explaining the links between risk factors.

We chose to consider all the previously identified cLBP risk factors and aimed to investigate their frequency and their interrelations with adapted multiple correspondence analysis in a French national sample of patients consulting their general practitioners (GPs) for cLBP.

## Methods

### Trial design

We conducted a 2-month prospective, multicenter, descriptive, cross-sectional, national survey.

### GP selection

We invited 3000 GPs selected at random from a national database (Logimed) of 20184 GPs to participate in the study.

### Patients

Each participating GP had to enroll at least one patient with cLBP within 2 months from the beginning of the study. The patients were seen during a routine visit to their GPs. LBP was defined as chronic when it lasted longer than 3 months. Patients were excluded if they a) were younger than 18 years or older than 60 years; b) had LBP for less than 3 months; c) had predominant sciatica; d) had back pain related to infection, tumor, or inflammatory disease; or d) were pregnant.

### Ethical approval

The study protocol was approved by the Commission Nationale Informatique et Liberté and the French National Medical Council (Conseil National de l'Ordre des Médecins). The study was conducted in compliance with the protocol Good Clinical Practices and Declaration of Helsinki principles. In accordance with French national law, GPs and patients gave their written agreement to participate after being informed about the study protocol.

### Intervention

The GPs completed 2 separate questionnaires.

### GP questionnaire

GPs completed a questionnaire asking about their demographic (age and sex) and professional data (area, urban or rural, and exclusively private or public/private practice).

### Patient questionnaire

GPs collected the following data about patients during the visit: demographics (age, sex and marital status), clinical characteristics (weight and height), and the presence of cLBP risk factors (medical, psychological, social and professional). Patients were interviewed about pain intensity at the onset of the current LBP episode (weak, moderate, severe, extremely severe), presence of sciatica at the onset of the current LBP episode (yes/no), initial limitation of activities of daily living (no limitation, moderate, severe, extremely severe), other types of musculoskeletal pain (yes/no), history of lumbar spine surgery (yes/no), duration of the current LBP episode (days), recurrent or previous history of LBP (yes/no), absence from work due to LBP before the current episode (yes/no), employment status (white- and blue-collar workers), job satisfaction (very satisfied, satisfied, poorly satisfied, unsatisfied), poor quality of relations with employer (yes/no), poor quality of relations with co-workers (yes/no), lack of recognition at work (yes/no), beliefs that professional activities were responsible for LBP (yes/no), beliefs that physical activities at work are dangerous for back (yes/no), beliefs that maintaining a specific posture at work is responsible for LBP (yes/no), frequent heavy lifting at work (yes/no), work-related injuries as a cause of pain (yes/no), litigation with health insurance organism (yes/no), education level (no full-time education, primary school, high school, post-graduate education), perceived inadequate income (yes/no), history of treated episode of anxiety (yes/no), history of treated episode of depression (yes/no), neurotic personality disorder (yes/no), poor general health status (yes/no), and medication intake for the previous week (analgesics, nonsteroidal anti-inflammatory drugs [NSAIDs], muscle relaxants, other).

### Sample size of patients

To calculate sample size, we considered the risk factor with the lowest prevalence in the studied population. Depression has previously been reported as having the lowest prevalence (8.7% to 10.2%) among all identified risk factors for cLBP [27]. We calculated a sample size of 3800 patients as being needed to estimate this proportion (p = 0.0875), with a 95% confidence interval and an absolute precision of 0.9% by use of the following formula: N = p(1−p) (1.96/precision)^2^, where precision of the estimate was 0.009.

### Statistical analysis

Data analysis involved use of SAS 8.2 software (SAS institute Inc, Cary, NC, USA). All quantitative variables was tested for normal distribution; those with normal distribution were described with means and 95% confidence intervals, and those with non-normal distribution were described with medians and 95% confidence intervals. Qualitative variables are described with proportions and percentages. The frequency of each cLBP risk factor and its 95% confidence interval were calculated for the whole sample and for subgroups of patients according to their professional status (working/nonworking) and duration of cLBP (≤2 years/>2 years). We chose the cut-off of 2 years' duration of cLBP in accordance with results of a previous study showing that for individuals disabled for longer than 2 years, the return-to-work rate is close to zero [2], [7].

We used multiple correspondence analysis (MCA) to study simultaneously the interrelations among the set of risk factors for cLBP [29].

1. Each risk factor was dichotomized into 2 modalities (presence = “yes”, absence = “no”) and each modality must be considered in the analysis as a separate variable. The items with multiple response categories were collapsed into dichotomous categories as follows: 1) for pain intensity at the onset of the current LBP episode, weak or moderate was considered as “no” and severe or extremely severe as “yes”; 2) for initial limitation of activities of daily living, no limitation or moderate were considered as “no” and severe or extremely severe as “yes”; 3) for job satisfaction, very satisfied or satisfied was considered as “no” and poorly satisfied or unsatisfied as “yes”; 4) for education level, high school or post-graduate education was considered as “no” and primary school or no full-time education as “yes”. Therefore, we obtained a cross-tabulation table with subjects as rows and modalities (“yes” and “no”) as columns. Thus, with N risk factors, a table having 2N columns was analyzed.
2. One aim of the method is to produce a map of this table with each column represented by a point. This approach is very similar to that of factor analysis in that a measure of total variance of the table is defined, and this total is decomposed optimally along the so-called principal axis (dimension). As with factor analysis, the proportion of the variance (inertia) explained by the main dimension (axis) is calculated, and the number of retained dimensions is chosen, by the scree test, to obtain a cumulative percentage of acceptable variance [30].
3. Several statistical parameters (contribution of each modality to each dimension, weights, coordinates, etc.) are calculated to characterize each modality.
4. The projections of the modalities are graphically represented as points in different planes formed by the main dimensions (ie, axes 1 and 2, axes 1 and 3, axes 2 and 3). Here, to simplify the presentation, only the projections on the plane formed by axes 1 and 2 are given. This graphic representation allows for visualizing the grouped (ie, associated) modalities and helps in the interpretation of dimensions (see figure S1 and its interpretation in the Results section).
5. This visual interpretation of the data is mathematically confirmed or not by using calculated parameters in step 3 above. With this process, one obtains the exact set of points that contribute strongly to the creation of a given dimension. The clinical study of this set of modalities allows for naming and interpreting medically the dimension (see examples in the Results section).

## Results

### Flow of participants through the trial

The Logimed database contains information on 20184 GPs. A total of 3000 GPs were selected at random from the database and asked to participate.

**GPs characteristics:** Of the GPs selected, 2847 (94.9%) agreed to participate, 2068 (68.9%) including at least 1 patient. The mean age was 48.0±6.9 years, 87.9% were male, and 62.9% worked in an urban environment.

**Patient characteristics:** A total of 7117 patients were interviewed by their GP, and the data for 4522 (63.5%) were analyzed. In total, 1197 (16.8%) patients were excluded because they were younger than 18 or older than 60 and 1398 (19.6%) because they had acute or sub-acute LBP (duration of pain less than 3 months).

The demographic and clinical characteristics of patients are shown in **Table 1**. Patients' mean age was 46.2±9.2 years; 57.2% were male, and 76.7% were working. The mean pain duration was 19.4±25.5 months, and 21.2% of patients had a LBP duration of more than 2 years. More than 90% of patients had taken analgesics, 57.0% NSAIDs and 47.6% muscle relaxants.

**Table 1:** Demographic and clinical characteristics of patients consulting their general practitioners for low back pain (LBP).

	Whole sample		LBP≤2 years	LBP>2 years
	(N = 4522)	Missing values	(N = 3563)	(N = 959)
Age, years (mean [95% CI])	46.2 [45.9 to 46.4]	0 (0)	45.8 [45.5 to 46.1]	47.6 [47.1 to 48.2]
Male, n (%)	2578 (57.2)	14 (0.3)	2018 (56.7)	560 (58.9)
Weight, kg (median [95% CI])	75 [65 to 83]	60 (1.3)	75 [66 to 85]	75 [65 to 83]
Height, cm (median [95% CI])	170 [164 to 176]	64 (1.4)	170.2 [164 to 176]	170.3 [164 to 176]
Body mass index, kg/m^2^ (median [95% CI])	25.4 [23.0 to 27.8]	71 (1.6)	25.3 [22.9 to 27.7]	25.7 [23.2 to 28.4]
Married, n (%)	3505 (83.4)	320 (7)	2766 (83.7)	739 (82.5)
Professional activity, n (%)	3469 (76.7)	0 (0)	2761 (77.5)	708 (73.8)
Back pain duration, months (mean [95% CI])	19.4 [18.6 to 20.1]	0 (0)	9.7 [9.5 to 9.9]	55.3 [53.1 to 57.6]
Education level, n (%)				
No full time education	122 (2.7)	36 (0.8)	88 (2.5)	34 (3.6)
Primary school	1512 (33.7)		1178 (33.4)	334 (35.0)
High school	1973 (44.0)		1567 (44.4)	406 (42.6)
Post graduate	879 (19.6)		699 (19.8)	180 (18.9)
Current medications, n (%)				
Analgesics	4246 (93.9)	0 (0)		
NSAIDs	2577 (57.0)			
Muscle relaxants	2151 (47.6)			
Other	223 (4.9)			
No treatment	99 (2.2)			

Note: Values are numbers (percentages), unless otherwise indicated.

CI: confidence interval.

NSAIDs: nonsteroidal anti-inflammatory drugs.

### Frequency of cLBP risk factors in the whole sample (N = 4522)

The frequency of medical, social, and psychological risk factors for cLBP in the whole sample is shown in **Table 2**. The highest frequencies were observed for history of recurrent LBP (72.1%), initial limitation of activities of daily living (66.4%), pain intensity at onset of the current episode (62.9%), absence from work due to LBP before the current episode (62.4%), and history of treated episode of anxiety (44.0%).

**Table 2:** Frequency [95% confidence interval] of nonprofessional risk factors for chronic low back pain (cLBP) for all patients who consult their general practitioners for LBP and subgroups of patients depending on duration of LBP.

Nonprofessional risk factors	All patients		Subgroups of cLBP patients	
	(N = 4522)	Missing values	LBP≤2 years	LBP>2 years
			(N = 3563)	(N = 959)
**Medical risk factors**				
History of recurrent LBP	72.1% [70.8 to 73.4]	0.5%	69.7% [68.1 to 71.2]	81.1% [78.5 to 83.6]*
Initial limitation of activities of daily living	66.4% [65.0 to 67.8]	0.3%	65.0% [63.4 to 66.6]	71.7% [68.7 to 74.5]*
Pain intensity at the onset of the current episode of LBP (severe or extremely severe)	62.9% [61.5 to 64.3]	0.1%	61.2% [59.6 to 62.8]	69.2% [66.2 to 72.2]*
Absence from work due to LBP before the current episode	62.4% [60.9 to 63.8]	0.6%	60.0% [58.4 to 61.6]	71.2% [68.2 to 74.1]*
Presence of sciatica at the onset of the current episode	38.6% [37.1 to 40.1]	6.8%	37.8% [36.2 to 39.5]	41.5% [38.2 to 44.8]
Other types of musculoskeletal pain	36.2% [34.7 to 37.7]	12.7%	33.8% [32.2 to 35.5]	45.1% [41.7 to 48.6]*
Poor general health status	15.8% [14.7 to 16.9]	2.9%	14.8% [13.6 to 16.0]	19.5% [17.0 to 22.2]*
History of lumbar spine surgery	12.3% [11.3 to 13.3]	0.5%	9.8% [8.8 to 10.8]	21.7% [19.1 to 24.4]*
**Psychological risk factors**				
History of treated episode of anxiety	44.0% [42.5 to 45.4]	0.3%	41.8% [40.2 to 43.5]	51.9% [48.7 to 55.1]*
History of treated episode of depression	27.0% [25.7 to 28.3]	0.2%	24.8% [23.4 to 26.3]	35.2% [32.2 to 38.3]*
Neurotic personality disorder	13.8% [12.8 to 14.8]	0.4%	12.5% [11.4 to 13.6]	18.7% [16.3 to 21.3]*
**Social risk factors**				
No full-time education or primary school only	36.4% [35.0 to 37.9]	0.8%	35.8% [34.3 to 37.5]	38.6% [35.5 to 41.7]
Perceived inadequate income	36.2% [34.7 to 37.6]	6.7%	35.6% [33.9 to 37.2]	38.4% [35.3 to 41.7]

* significant difference P<0.05 in frequency of risk factor between the two subgroups of patients.

### Frequency of professional cLBP risk factors for working patients (N = 3469)

Working patients had a mean frequency of 9.2±4.0 of 22 cLBP risk factors. The frequency of professional cLBP risk factors for working patients is shown in **Table 3**. The highest frequencies were observed for beliefs, that LBP is due to maintaining a specific posture at work (79.0%) and professional activities (64.4%) and that physical activities at work are dangerous for the lower back (62.0%); frequent heavy lifting at work (60.5%) and lack of recognition at work (49.3%) were also frequently cited.

**Table 3:** Frequency [95% confidence interval] of professional risk factors for chronic low back pain (cLBP) for working patients who consult their general practitioners for LBP and subgroups of patients depending on duration of LBP.

Professional risk factors	Working patients		Subgroups of cLBP patients	
	(N = 3469)	Missing values	LBP≤2 years	LBP>2 years
			(N = 2761)	(N = 708)
Beliefs that maintaining specific postures at work is responsible for LBP	79.0% [77.6 to 80.3]	0.3%	78.7% [77.1 to 80.2]	80.2% [77.0 to 83.1]
Beliefs that professional activities are responsible for LBP	65.4% [63.8 to 67.0]	0.6%	64.9% [63.1 to 66.7]	67.3% [63.7 to 70.8]
Beliefs that physical activities are dangerous for the lower back	62.0% [60.4 to 63.6]	0.3%	61.4% [59.6 to 63.2]	64.4% [60.7 to 67.9]
Frequent heavy lifting at work	60.5% [58.9 to 62.2]	0.3%	60.8% [58.9 to 62.6]	59.7% [56.0 to 63.3]
Lack of recognition at work	49.3% [47.6 to 51.0]	0.9%	49.1% [47.3 to 51.0]	50.0% [46.2 to 53.8]
Poor or no satisfaction with job	40.6% [39.0 to 42.3]	0.5%	39.1% [37.3 to 41.0]	46.4% [42.7 to 50.2]*
Poor quality of relations with employer	27.7% [26.2 to 29.3]	1.9%	27.1% [25.4 to 28.8]	30.3% [26.9 to 33.9]
Poor quality of relations with co-workers	15.0% [13.8 to 16.2]	1.6%	14.8% [13.5 to 16.2]	15.7% [13.1 to 18.7]
Work-related injury as the cause of pain	13.0% [11.9 to 14.2]	0.7%	12.7% [11.5 to 14.0]	14.3% [11.8 to 17.1]

* significant difference p<0.05 in frequency of risk factors between the two subgroups of patients.

### Frequency of risk factors depending on length of LBP

Patients with cLBP for more than 2 years were slightly older than those with cLBP for 2 years or less (47.6±8.5 vs 45.8±9.3; **Table 1**). Medical, psychological, and social risk factors for cLBP tended to be more frequent the longer the duration of cLBP (**Table 2**). However, the frequency of professional cLBP risk factors seemed to be comparable regardless of length of cLBP (**Table 2**), except for “poor or no satisfaction with job” with a high frequency for patients with greater than 2 years' duration of cLBP (**Table 3**).

### Multiple Correspondence Analysis

#### Working patients (N = 3469)

We identified 3 main dimensions (axes), which explained 38.9% of the variance among 22 professional and nonprofessional risk factors for cLBP. The percentages of variance (inertia) explained by each dimension are given in **Table 4**: dimension 1 explained 19.4% of the total variance, and dimensions 2 and 3 explained 11.0% and 8.5%, respectively, of the variance.

**Table 4:** Multiple correspondence analysis of risk factor sets of the principal dimensions for working patients consulting their general practitioners for low back pain (LBP). List of risk factors with the best contribution to each determined dimension.

	DIMENSION 1 “ work-related”	DIMENSION 2 “psychological”	DIMENSION 3 “health-related”
**Variance (percentage)**	19.4	11.0	8.5
**Risk Factors**	Job satisfaction (Q10)	History of treated episode of anxiety (Q25)	Pain intensity at onset of the current episode of LBP (Q1)
	Recognition at work (Q13)	History of treated episode of depression (Q24)	Initial limitation of activities of daily living (Q3)
	Beliefs that professional activities are responsible for LBP (Q14)	Poor quality of relations with employer (Q11)	Presence of sciatica at onset of the current episode (Q2)
	Beliefs that physical activities are dangerous for the lower back (Q15)	Poor quality of relations with co-workers (Q12)	History of recurrent LBP (Q7)
	Beliefs that maintaining specific postures at work is responsible for LBP (Q16)	Neurotic personality disorder (Q26)	Absence from work due to LBP before the current episode (Q8)
	Frequent heavy lifting at work (Q17)	Other types of musculoskeletal pain (Q4)	History of lumbar spine surgery (Q5)
	Work-related injury as the cause of pain (Q18)		
	No full-time education or primary school only (Q20)		
	Perceived inadequate income (Q22)		

The projections of the modalities (“yes” and “no”) for each risk factor are represented as points in 3 planes formed by the main dimensions: axes 1 and 2 (Figure S1), axes 1 and 3, and axes 2 and 3.

#### Interpretation of Figure S1

See appendix S1: question details (Q1, Q2, etc.). In the upper left quadrant of the graph, the modalities “no” for Q16, Q14, Q15 and Q17 are far from the origin and close to each other, which suggests first that all these modalities contribute to the variance of axis 1 and thus to its construction and second, that the 4 modalities are associated. The study of the statistical parameters (contribution to each axis, coordinates, etc.) calculated for each of the modalities confirms, this time reliably and mathematically, this visual interpretation: the set of these 4 modalities contribute strongly to dimension 1.

Using the same method, visual interpretation then mathematical verification, we found that Q10 “no” and Q13 “no” are associated and both contribute to axis 1.

Contrary to the visual impression (these points are closer to the origin than the 4 previous points for dimension 1), mathematical verification revealed that among all the modalities, these points had maximal contribution to dimension 1.

Q18 “no,” Q20 “no,” and Q22 “no” also contribute to dimension 1.

The modalities “yes” for the 9 previous risk factors with “no” modalities (Q10, Q13, Q14, Q15, Q16, Q17, Q18, Q20, Q22) also contribute to dimension 1.

The interpretation (visual, then mathematic) of the projections of all the modalities onto the plane defined by axes 1 and 3 and that formed by axes 2 and 3 confirms the previous findings. To simplify the presentation, the figures for these 2 planes are not given.

Because the modalities “yes” and “no” of the 9 risk factors studied above both contribute to dimension 1, these factors are listed in **Table 4** without distinguishing the 2 modalities for the same factor.

**Table 4** shows that dimension 1 comprised all the professional risk factors, except for “relations with employer” and “relations with co-workers.” In addition, this dimension included social risk factors (level of education, perceived inadequate income). Thus, dimension 1 was interpreted as the “work-related” dimension. Dimension 2 comprised all the psychological risk factors. In addition, this dimension included “relations with employer,” “relations with co-workers,” and “other types of musculoskeletal pain”. Thus, dimension 2 was interpreted as the “psychological” dimension. Dimension 3 grouped all the medical risk factors and was interpreted as the “health-related” dimension.

#### Nonworking patients

We retained 3 main dimensions (axes) from the 13 nonprofessional risk factors, which explained 43.7% of the variance in cLBP for nonworking patients. The proportion of variance (inertia) explained by each dimension is given in **Table 5**. Dimension 1 explained 19.2% of the total variance and included one risk factor (“poor general health status”). Thus, dimension 1 was interpreted as the “general health status” dimension. Dimension 2 explained 14.2% of the total variance and comprised all the psychological risk factors and the “initial limitation of activities of daily living (ADL)” factor. Thus, dimension 2 was interpreted as the “psychological” dimension. Dimension 3 explained 10.2% of the total variance and comprised all the medical risk factors, except “poor general health status” and “presence of sciatica at the onset of the current episode.” Thus, this dimension was interpreted as the “medical” dimension. **Table 5** gives the risk factors contributing the most to each dimension. **Figure S2** represents, for the nonworking patients, the 2 modalities “yes” and “no” for each nonprofessional risk factor for the dimensions “poor general health status” (dimension 1) and “psychological” (dimension 2).

**Table 5:** Multiple correspondence analysis of risk factor sets of the principal dimensions for nonworking patients consulting their general practitioners for low back pain (LBP). List of risk factors with the best contribution to each determined dimension.

	DIMENSION 1 “general health status”	DIMENSION 2 “psychological”	DIMENSION 3 “medical”
**Variance (percentage)**	19.24	14.21	10.21
**Risk Factors**	Poor general health status (Q27)	History of treated episode of anxiety (Q25)	Pain intensity at onset of the current episode of LBP (Q1)
		Initial limitation of activities of daily living (Q3)	Absence from work due to LBP before the current episode (Q8)
		History of treated episode of depression (Q24)	Other types of musculoskeletal pain (Q4)
		Neurotic personality disorder (Q26)	History of recurrent LBP (Q7)

## Discussion

This cross-sectional national study in a large sample of cLBP patients in primary care confirmed a high frequency of previously identified risk factors, which suggests that our sample resembles those previously reported on this topic. The strength of this study is the variety of risk factors addressed and the use of MCA, which allows for analyzing the interrelations among these risk factors by defining dimensions of risk factors for cLBP and determining the contribution of each risk factor to the dimensions. To our knowledge, very few surveys examined the interrelation of identified cLBP risk factors and evaluated the contribution of risk factors to professional, medical and psychological dimensions of cLBP [31].

The literature on risk factors for cLBP is abundant, but numerous prospective studies assessed only a specific category of cLBP risk factors (professional, psychological or medical). These studies give only limited information because they do not allow for 1) analyzing the structure of the existing relations between all the risk factors or 2) discovering the underlying dimensions explaining the interfactor links. For example, in the prospective study of Valat et al. [18], which is methodologically valid, the authors selected explicit risk factors using only statistical criteria. Thus, they did not (wrongly) [29] take into account an important clinical factor “satisfaction with professional activity” because it was not found to be statistically significant. Moreover, no psychological factor was studied to explain “chronicity”. This study, although methodologically valid, does not take into account several risk factors previously identified.

The strength of the MCA analysis was its ability to examine the relevant importance of work-related factors in the working population as compared with psychological and other social factors. Indeed, MCA analysis revealed that the “work-related” dimension was the most important for patients with cLBP. Poor job satisfaction and lack of recognition at work contributed largely to this dimension, which suggests that “social work-related” factors probably weigh more than “physical work-related” ones. Moreover, patients with more than 2 years' duration of cLBP tended to report dissatisfaction with their jobs more often than those with 2 years' or less duration. Our results are in agreement with other studies showing poor job satisfaction and lack of recognition associated with cLBP [12], [14], [15], [17], [31].

Among professional factors, beliefs about the harmfulness of posture and physical activities as being responsible for cLBP were frequently cited and largely contributed to the “work-related” dimension. These results are in accordance with those from an increasing number of studies concerning the influence and consequences of pain-related fears and associated avoidance behavior in the development and maintenance of disabling LBP [32]–[34]. Self-reported feelings of disability and irrational and/or negative beliefs about pain such as kinesiophobia and fear avoidance have been associated with chronic evolution of LBP [35]–[37]. This the first report comparing the contribution of these risk factors with other risk factors.

As expected, a history of anxiety and depression largely contributed to the “psychological” dimension. Relationships with employers and co-workers, categorized as professional factors, also contributed to this dimension. Indeed, these variables could reflect more general behavioral attitudes with others than specific work-related attitudes.

The “health-related” dimension was the least important in this sample. This dimension concerned previously identified medical risk factors such as pain intensity or presence of sciatica at the onset of the current episode of LBP, initial limitation of ADL, history of recurrent LBP, absence from work due to LBP before the current episode and history of lumbar spine surgery.

For the nonworking patients, MCA revealed that GPs' poor opinion of their patients' general health status represents a dimension by itself. Poor general health status has already been reported as a risk factor of severity in several pathologic situations [10], but this is the first report to describe the contribution of this risk factor in terms of other risk factors. As was observed for working patients, for nonworking patients, the second and third dimensions were the “psychological” and “health-related” dimensions, with history of anxiety and depression largely contributing to the “psychological” dimension.”

Our study contains a number of limitations. First, the study was cross-sectional and the positive associations found do not allow for inferring causation. However, the pre-selected risk factors of chronicity were those most often identified in previous studies of risk factors for patients with LBP [2], [9], [10]–[20]. Second, the participating GPs may have failed to include all the referred patients, possibly creating a selection bias. Third, a retrospective study of subjects who are feeling pain for a long time may not provide reliable data about psychological states and affects before the onset of pain. Fourth, we used a pragmatic approach for collecting risk factors (risk factors were assessed by GPs, and questions with simple yes/no answers were used); more comprehensive assessments were not possible because of the large sample size. Fifth, the interrelations presented reflect less than 50% of the total variance. Finally, our results are mainly biostatistically based but the clinical application is substantial.

In conclusion, our results shed light on the interrelation and respective contribution of several previously identified risk factors for cLBP. They suggest that risk factors representing a “work-related” dimension are the most important risk factors for cLBP in the working population. Among these factors, patients' job satisfaction and job recognition largely contribute to this dimension and must be considered in prospective studies. Such feelings about professional conditions in LBP patients should be systematically recorded and taken into account by professionals. As previously recommended by the European guidelines (COST B13) for the management of LBP, educational and behavioral therapy programs on these topics should be proposed and evaluated in cLBP [38].

## Supporting Information

Figure S1 Multiple correspondence analysis of working patients consulting their general practitioners for chronic low back pain. The two principal retained dimensions (work-related and psychological) are represented in this figure. Each risk factor was dichotomized in 2 modalities (presence = yes, absence = no). The 4 red circumferences contain the “yes” and “no” modalities that strongly contribute to dimension 1. See appendix S1 for question details (Q1, Q2, etc.).
(0.10 MB DOC)

Figure S2 Multiple correspondence analysis of nonworking patients consulting their general practitioners for chronic low back pain. The two principal retained dimensions (general health status and psychological) are represented in this figure. Each risk factor was dichotomized in 2 modalities (presence = yes, absence = no). See appendix S1 for question details (Q1, Q2, etc.).
(0.08 MB DOC)

Appendix S1 Question details
(0.02 MB DOC)
