# Supplementary material for: Frequency and Interrelations of Risk Factors for Chronic Low Back Pain in a Primary Care Setting
Source: PLoS One. 2009 Mar 16;4(3):e4874. doi: 10.1371/journal.pone.0004874 (PMC2654108; doi:10.1371/journal.pone.0004874)
Supplement: Figure S1 — Multiple correspondence analysis of working patients consulting their general practitioners for chronic low back pain. The two principal retained dimensions (work-related and psychological) are represented in this figure. Each risk factor was dichotomized in 2 modalities (presence = yes, absence = no). The 4 red circumferences contain the “yes” and “no” modalities that strongly contribute to dimension 1. See appendix S1 for question details (Q1, Q2, etc.). (0.10 MB DOC) [file pone.0004874.s001.doc]

**Figure S1****.**
