# Supplementary material for: Frequency and Interrelations of Risk Factors for Chronic Low Back Pain in a Primary Care Setting
Source: PLoS One. 2009 Mar 16;4(3):e4874. doi: 10.1371/journal.pone.0004874 (PMC2654108; doi:10.1371/journal.pone.0004874)
Supplement: Appendix S1 — Question details (0.02 MB DOC) [file pone.0004874.s003.doc]

**Appendix S1 : question details**

Q1: Pain intensity at the onset of the current episode of LBP.

Q2: Presence of sciatica at the onset of the current episode

Q3: Initial limitation of activities of daily living

Q4: Other types of musculoskeletal pain

Q5: History of lumbar spine surgery.

Q7: History of recurrent LBP.

Q8: Absence from work due to LBP before the current episode.

Q10: Job satisfaction.

Q11: Poor quality of relations with employer.

Q12: Poor quality of relations with co-workers.

Q13: Recognition at work.

Q14: Beliefs that professional activities are responsible for LBP.

Q15: Beliefs that physical activities are dangerous for the lower back.

Q16: Beliefs that maintaining specific postures at work is responsible for LBP.

Q17: Frequent heavy lifting at work.

Q18: Work-related injury as the cause of pain.

Q20: No full-time education or primary school only.

Q22: Perceived inadequate income.

Q24: History of treated episode of depression.

Q25: History of treated episode of anxiety.

Q26: Neurotic personality disorder.

Q27: Poor general health status
